# Cognitive Profile in Adult Patients With Myelin Oligodendrocyte Glycoprotein Antibody‐Associated Disease: A Comparative Study With Multiple Sclerosis

**DOI:** 10.1111/ene.70115

**Published:** 2025-03-19

**Authors:** Giorgia Teresa Maniscalco, Antonio Rosario Ziello, Elisa Mantovani, Alessandro Dinoto, Daniele Di Giulio Cesare, Ornella Moreggia, Maria Elena Di Battista, Sara Carta, Vanessa Chiodega, Emanuela Stoppele, Sergio Ferrari, Vincenzo Andreone, Stefano Tamburin, Sara Mariotto

**Affiliations:** ^1^ Multiple Sclerosis Center, “A. Cardarelli Hospital” Naples Italy; ^2^ Neurological Clinic and Stroke Unit A. Cardarelli Hospital Naples Italy; ^3^ Neurology Section Department of Neurosciences, Biomedicine, and Movement Sciences, University of Verona Verona Italy

**Keywords:** cognition, multiple sclerosis (MS), myelin oligodendrocyte glycoprotein antibody‐associated disease (MOGAD), neuropsychological battery

## Abstract

**Background:**

Myelin oligodendrocyte glycoprotein antibody‐associated disease (MOGAD) has emerged as an acquired immune‐mediated demyelinating disorder of the central nervous system distinct from multiple sclerosis (MS). Cognitive dysfunction and related symptoms (anxiety, depression, fatigue) and their impact on quality of life (QoL) have been in‐depth characterized in MS, but data in adult MOGAD patients are very preliminary.

**Methods:**

This study aims to characterize cognitive changes through an extensive cognitive battery, as well as anxiety, depression, fatigue, and QoL, in adult MOGAD compared to MS patients.

**Results:**

Cognitive outcomes (number of patients with abnormal scores, score severity) depression, anxiety, fatigue, and QoL were largely comparable between MOGAD and MS patients. Most cognitive outcomes were not significantly correlated with neuropsychiatric symptoms, fatigue, and QOL in MOGAD.

**Conclusions:**

Our study underscores the importance of cognitive and related outcomes in MOGAD patients and the need for future studies exploring their pathophysiological and cortical morphometric underpinnings and potential therapeutic approaches.

## 1 Introduction

Myelin oligodendrocyte glycoprotein antibody‐associated disease (MOGAD) is an antibody‐mediated disorder with a relapsing course in half of cases and disability determined by sequelae of clinical attacks [1]. Current evidence does not support a relapse‐independent activity or a progressive course in MOGAD, at variance with multiple sclerosis (MS) [2, 3].

Cognitive dysfunction plays a key role in MS, and it has been in‐depth characterized [4]. On the contrary, cognitive function in MOGAD has been explored in few studies, including mainly pediatric patients, in whom acute disseminated encephalomyelitis (ADEM) is the most common clinical phenotype and is associated with impaired brain development [5]. Apart from a recently published prospective multicentre study [6], cognitive outcomes in adults with MOGAD are limited to case reports or small cohorts. Of note, in MS patients, cognitive impairment is often associated with neuropsychiatric symptoms (anxiety, depression) and fatigue but not with motor and other deficits, suggesting specific pathophysiological mechanisms for this symptoms cluster that negatively affect quality of life (QoL) [7]. Literature on neuropsychiatric symptoms, fatigue, and QoL is limited in MOGAD [6, 8, 9, 10].

Our primary aim is to characterize cognitive changes in adults with MOGAD through a standardized cognitive battery and to compare the results with a control cohort of MS patients matched for demographic and clinical features. The secondary aims were to compare anxiety, depression, fatigue, and QoL in MOGAD versus MS patients and to explore their correlation with cognitive measures in MOGAD.

## 2 Methods

### 2.1 Study Design

This observational, prospective, multi‐center, cross‐sectional, case–control study recruited patients with MOGAD and relapsing–remitting MS at the Multiple Sclerosis and Neuroimmunology Center, Antonio Cardarelli Hospital Naples, and the Neurology Section, Department of Neurosciences, Biomedicine, and Movement Sciences, University of Verona, between December 2022 and September 2023. All patients signed an informed consent for the study that was conducted according to the Declaration of Helsinki and approved by the Ethical Committee Campania 3 (approval # AORN 063).

Inclusion criteria: (a) diagnosis of MOGAD according to the International MOGAD panel criteria [11] or relapsing–remitting MS according to the 2017 McDonald diagnostic criteria [12]; (b) disease duration ≥ 5 years; (c) age ≥ 18 years. Exclusion criteria: (d) any relapse and/or steroid use in the 30 days before enrollment; (e) severe systemic disease or neoplasms; (f) psychiatric disorders, preexisting cognitive deterioration, or intellectual disability; and (g) active/previous alcohol or substance use disorder.

### 2.2 Neuropsychological Assessment

Patients with MOGAD and MS underwent a full neuropsychological battery, which was performed by two trained neuropsychologists using the Brief Repeatable Battery of Neuropsychological Tests (BRB‐N, version A [13]) and the Stroop Test [14]. The BRB‐N includes tests of verbal learning (selective reminding test long‐term storage, SRT‐LTS; selective reminding test consistent long‐term retrieval, SRT‐CLTR) and memory (selective reminding test delayed recall, SRT‐D), visual/spatial learning and memory (10/36 spatial recall test, SPART) and its delayed recall (SPART‐D), complex attention and information processing speed (symbol digit modalities test, SDMT; paced auditory serial addition test 2″ and 3″, PASAT) and verbal fluency to semantic stimuli (word list generation, WLG). The Stroop Test evaluates executive functioning (cognitive interference inhibition).

*Z*‐scores for all BRB‐N subtests were calculated using Italian normative data (mean, standard deviation, SD) [13]. Raw scores of the Stroop Test were corrected for age and education according to Italian normative data, whose distribution was non‐parametric [14]. Cognitive performance was considered abnormal if individual *Z*‐ or corrected scores ≤ −1.5 SDs of the normative values or the cut‐off (fifth percentile), respectively.

### 2.3 Anxiety, Depression, Fatigue and QoL Measures

Anxiety, depression, fatigue, and QoL were assessed with the Hospital Anxiety and Depression Scale (HADS [15]), the Modified Fatigue Impact Scale (MFIS^s16^) and the European Quality of Life‐5 Dimensions questionnaire (EQ‐5D; index score, visual analogue scale, VAS^s17^), respectively.

### 2.4 Statistics

Statistical analysis was performed with SPSS version 21.0 (SPSS, Chicago, USA). For continuous variables, normality of distribution was tested with the Shapiro–Wilk test, and an independent samples *t*‐test was used for normal distribution and *Z*‐scores, while the non‐parametric Mann–Whitney *U*‐test was applied to non‐parametric distributions. The chi‐squared test, with Yates' correction when needed, was applied to dichotomous variables. Correlations were explored with the non‐parametric Spearman's *ρ* correlation coefficient. *p* < 0.05 (two‐tailed) was the significance threshold for all the tests.

## 3 Results

We included 19 MOGAD and 19 MS patients, with comparable baseline demographic and clinical characteristics (Table 1).

**TABLE 1:** Demographic and clinical features of the included patients.

	MOGAD (*N* = 19)	MS (*N* = 19)	*p*
Demographic			
Women/men	10/9	12/7	0.51
Age, years	41.3 (18.5), 42, 24–56.5	38.8 (9.8), 38, 31–46	0.62
Education, years	10.4 (3.7), 8, 8–13	11.5 (4.1), 13, 8–13	0.28
Clinical			
Disease duration, mos	22.0 (13.2), 22, 11.5–34	22.8 (12.6), 18, 15–27.5	0.89
Symptom(s) at onset			
Optic nerve	7	7	0.71
Spinal cord	6	8	
Brain/cerebellum	6	4	
EDSS score	2.1 (2.1),1.5, 0.5–2.8	2.1 (1.6), 2, 1–2.8	0.82
Active lesions, yes/no	9/10	5/14	0.18
Spinal cord lesions, yes/no	9/10	10/9	0.75
Brain lesions, yes/no	11/8	19/0	N.A.
DMT duration, mos	12.8 (12.0), 11, 2–19.5	19.2 (14.4), 15, 11.5–25.5	0.16

*Note:* Data are presented as *N* or mean (standard deviation), median, interquartile range.

Abbreviations: DMT, disease modifying therapy; EDSS, expanded disability status scale; MOGAD, myelin oligodendrocyte glycoprotein antibody‐associated disease; mos, months; MS, multiple sclerosis; N.A., not applicable.

**FIGURE 1:**
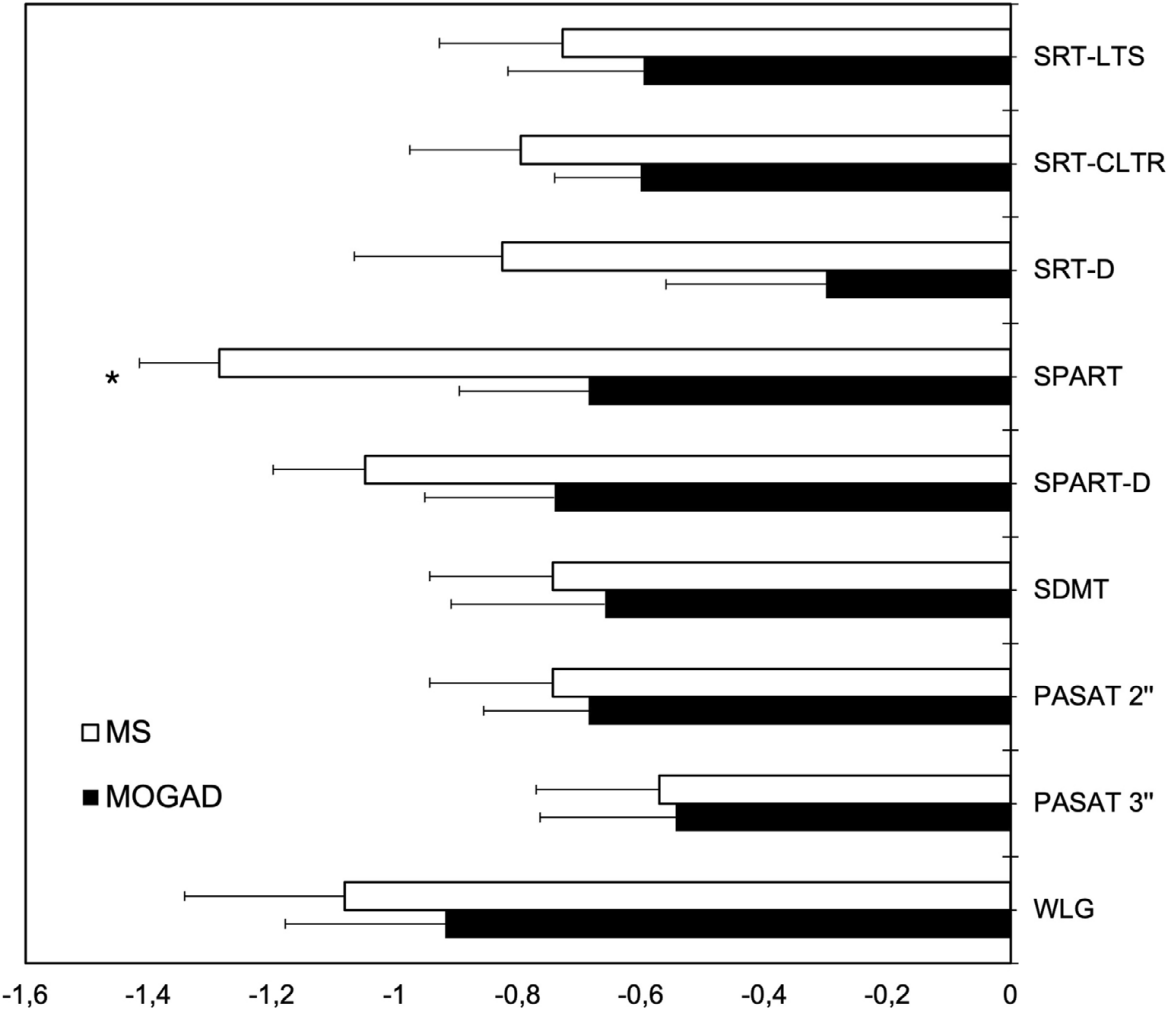
*Z*‐scores of the Brief Repeatable Battery of Neuropsychological Tests in patients with multiple sclerosis (MS) and myelin oligodendrocyte glycoprotein antibody‐associated disease (MOGAD). SDMT, symbol digit modalities test; SPART, 10/36 spatial recall test; SPART‐D, 10/36 spatial recall test delayed recall; SRT‐D, selective reminding test delayed recall; SRT‐CLTR, selective reminding test consistent long‐term retrieval; SRT‐LTS, selective reminding test long‐term storage; WLG, word list generation. *marks *p* < 0.05 for MS versus MOGAD comparison.

BRB‐N *Z*‐scores were overall more severe in MS than in MOGAD patients, but the difference was significant only for the SPART (*p* = 0.02), while the other outcomes were similar in the two groups (Table S1, Figure 1). The number of patients with abnormal BRB‐N *Z*‐scores (i.e., ≤ −1.5) did not significantly differ between the two groups (Chi‐squared test, *p* = 0.15–1, n.s.).

Stroop Test scores were similar in the two groups (Table S1), with 1/19 MS patients and 0/19 MOGAD patients showing abnormal Stroop Test times.

Anxiety, depression, fatigue, and QoL scores did not significantly differ between the two groups (Table S1).

Among neuropsychological outcomes, BRB‐N SDMT *Z*‐score was significantly correlated with HADS depression score (*ρ* = −0.49, *p* = 0.035) and EQ‐5D VAS (*ρ* = 0.47, *p* = 0.043), while the Stroop Test time was significantly correlated with EQ‐5D index score (*ρ* = −0.48, *p* = 0.039). The remaining correlations between cognitive and other outcomes were not significant in the MOGAD cohort.

## 4 Discussion

This study provides a detailed cognitive profile of adults with MOGAD and shows that (a) cognitive outcomes (number of patients with abnormal scores, score severity) did not significantly differ between matched MOGAD and MS patients, except for the SPART score that was significantly worse in MS patients; (b) depression, anxiety, fatigue, and QoL did not differ between groups; (c) most cognitive outcomes, except SDMT and Stroop test time, were not significantly correlated with neuropsychiatric symptoms, fatigue, and QoL in MOGAD patients.

Apart from case reports/series, only two studies applied extensive neuropsychological testing to adults with MOGAD. A “gray literature” retrospective chart review of nine patients reported impairment in at least one cognitive domain, mainly learning new information and executive function (lexical and semantic fluency) in 44% of cases^s18^. Semantic fluency deficits, along with worse visuomotor processing speed, were confirmed in a prospective, longitudinal, multicenter study including 113 patients with MOGAD compared with healthy controls. Cerebral lesions were correlated with neuropsychological deficits, and no decline in cognition was observed during the 2‐year follow‐up [6].

Our results suggest that MOGAD patients show overall poor performance in almost all cognitive domains, with executive function (semantic fluency) being more involved and a similar impairment compared to patients with MS in many cognitive domains. These results are relevant, as previous literature was scant and controversial, with some case series reporting overlapping cognitive measures between healthy controls and MOGAD patients^s19^ and other reports supporting the frequent occurrence of cognitive impairment in MOGAD [6],^s20^.

Several pieces of evidence pointed to gray matter (GM) and white matter (WM) damage, leading to neurodegeneration and disconnection, respectively, as key determinants of cognitive impairment in MS^s21^. Cognitive dysfunction in MS is associated with severe widespread cortical (e.g., frontal and temporal cortices) and subcortical (e.g., caudate, thalamus, and putamen) GM atrophy^s22^ and WM damage to relevant (e.g., corpus callosum and forceps minor/major) cortico‐subcortical connections and tracts^s23^.

The underlying anatomical correlates of cognitive decline in MOGAD are less defined and probably related to the atrophy of specific areas, such as hippocampal‐parahippocampal involvement^s24^ or cortical thinning of the pericalcarine, orbitofrontal, and temporal lobes^s25^. The potential involvement of the corpus callosum and thalamus may also account for cognitive decline, even though, for example, callosal lesions were not associated with disconnection syndrome^s26^. The rapidly evolving dynamics of MOGAD lesions and their resolution over time are a significant challenge to clearly identify the anatomical correlates of cognitive impairment in MOGAD^s27^.

We found similar anxiety, depression, fatigue, and QoL outcomes in MOGAD and MS patients, adding to the limited literature on this topic [6, 8, 9, 10],^s19^. Depression scores and QoL were negatively correlated with information processing speed and executive function, respectively, in MOGAD. Significant associations between depression and information processing speed decline over time,^s28^ and between specific aspects of cognitive impairment and QoL^s29^ have been reported in MS. Further research should better define whether, similarly to MS, cognitive, neuropsychiatric symptoms, and fatigue represent a symptoms cluster in MOGAD with underlying pathomechanisms that should be specifically addressed.

Strengths of this study are (a) the administration of a comprehensive neuropsychological battery, (b) the assessment of neuropsychiatric, fatigue, and QoL measures that are associated with cognition, and (c) the inclusion of a control group of patients with MS, a disease in which the cognitive profile has been extensively studied [4]. Limitations include (a) the small sample size, which mirrors the epidemiology of MOGAD and highlights the importance of multicenter studies, (b) the assessment of the most common neuropsychiatric symptoms only, and (c) the lack of neuroimaging data that impeded exploring a correlation between cognitive and gray matter/cortical changes.

Our study provides a comprehensive characterization of neuropsychological features in a cohort of patients with MOGAD, demonstrating poor cognitive performances and widespread neuropsychiatric abnormalities affecting quality of life. Our results have implications for the management of these conditions (discussing prognosis and sharing treatment decisions) and are relevant to our understanding of the disease.

## Author Contributions

**Giorgia Teresa Maniscalco:** conceptualization, methodology, data curation, writing – original draft. **Antonio Rosario Ziello:** conceptualization, methodology, data curation, writing – original draft. **Elisa Mantovani:** methodology, data curation, writing – original draft. **Alessandro Dinoto:** methodology, data curation, writing – review and editing. **Daniele Di Giulio Cesare:** methodology, data curation, writing – review and editing. **Ornella Moreggia:** methodology, data curation, writing – review and editing. **Maria Elena Di Battista:** methodology, data curation, writing – review and editing. **Sara Carta:** methodology, data curation, writing – review and editing. **Vanessa Chiodega:** methodology, data curation, writing – review and editing. **Emanuela Stoppele:** methodology, data curation, writing – review and editing. **Sergio Ferrari:** methodology, data curation, writing – review and editing. **Vincenzo Andreone:** methodology, data curation, writing – review and editing. **Stefano Tamburin:** conceptualization, data curation, writing – original draft, writing – review and editing, supervision. **Sara Mariotto:** conceptualization, data curation, writing – original draft, writing – review and editing, supervision.

## Ethics Statement

The study was conducted according to the Declaration of Helsinki and approved by the Ethical Committee Campania 3 (Approval # AORN 063).

## Conflicts of Interest

The authors declare no conflicts of interest.

## Supporting information


Data S1.

## Data Availability

The data that support the findings of this study are available from the corresponding author upon reasonable request.
