# Supplementary material for: Cognitive Profile in Adult Patients With Myelin Oligodendrocyte Glycoprotein Antibody‐Associated Disease: A Comparative Study With Multiple Sclerosis
Source: Eur J Neurol. 2025 Mar 19;32(3):e70115. doi: 10.1111/ene.70115 (PMC11921136; doi:10.1111/ene.70115)
Supplement: Supplementary file 1 — Data S1. [file ENE-32-e70115-s001.docx]

**SUPPLEMENTARY MATERIAL**

**Cognitive profile in adult patients with myelin oligodendrocyte glycoprotein antibody-associated disease: a comparative study with multiple sclerosis**

Giorgia Teresa Maniscalco^1,2*^, Antonio Rosario Ziello^1*^, Elisa Mantovani^3§^, Alessandro Dinoto^3^, Daniele Di Giulio Cesare^1^, Ornella Moreggia^1^, Maria Elena Di Battista^1,2^, Sara Carta^3^, Vanessa Chiodega^3^, Emanuela Stoppele^3^, Sergio Ferrari^3^, Vincenzo Andreone^2^, Stefano Tamburin^3#^, and Sara Mariotto^3#^

^1^Multiple Sclerosis Center, “A. Cardarelli Hospital”, Naples, Italy

^2^Neurological Clinic and Stroke Unit, “A. Cardarelli Hospital”, Naples, Italy

^3^Neurology Section, Department of Neurosciences, Biomedicine, and Movement Sciences, University of Verona, Verona, Italy

* These authors share first authorship

^#^ These authors share senior authorship

**^§^ Corresponding author.** Elisa Mantovani, MPsych, PhD, Department of Neurosciences, Biomedicine and Movement Sciences, Neurology Section, University of Verona, Piazzale Scuro 10, I-37134 Verona, Italy; Tel.: +39-347-630-5905; Fax: +39-045-802-7276; Email address: elisa.mantovani@univr.it; ORCID: 0000-0003-3717-7697.

Summary:

Supplementary table 1

Supplementary references

Supplementary table 1. Neuropsychological and other related outcomes

|  | **MOGAD (N = 19)** | **MS (N = 19)** | ***P*** |
| --- | --- | --- | --- |
| Brief repeatable battery of neuropsychological tests^§^ | | | |
| SRT-LTS | -0.60 (0.94); 2/17 | -0.73 (0.89); 5/14 | 0.66 |
| SRT-CLTR | -0.60 (0.63); 1/18 | -0.80 (0.77); 5/14 | 0.40 |
| SRT-D | -0.30 (1.12); 5/14 | -0.83 (1.05); 6/13 | 0.14 |
| SPART | -0.69 (0.90); 3/16 | -1.29 (0.58); 8/11 | 0.02* |
| SPART-D | -0.74 (0.93); 5/14 | -1.05 (0.67); 4/15 | 0.25 |
| SDMT | -0.66 (1,10); 4/15 | -0.74 (0.88); 3/16 | 0.80 |
| PASAT 2’’ | -0.69 (0.75); 2/17 | -0.74 (0.89); 4/15 | 0.92 |
| PASAT 3’’ | -0.54 (0.95); 2/17 | -0.57 (0.88); 4/15 | 0.93 |
| WLG | -0.92 (1.15); 4/15 | -1.08 (1.11); 3/16 | 0.66 |
| Stroop test^†^ | | | |
| Time | 18.8 (7.6), 19.5, 13-24.5; 0/19 | 18.5 (6.8), 16, 14.5-20.5; 1/19 | 0.89 |
| Errors | 0.47 (0.94), 0, 0-0.5; 0/19 | 0.47 (0.79), 0, 0-1; 0/19 | 0.99 |
| Anxiety and depression^‡^ | | | |
| HADS anxiety | 7.1 (3.4), 8.5, 5-11 | 9.4 (4.4), 9, 5-14 | 0.12 |
| HADS depression | 3.8 (3.1), 3, 2-5 | 3.7 (2.8), 3, 1-6 | 0.87 |
| Fatigue^‡^ | | | |
| MFIS physical | 15.0 (10.0), 14, 7-24 | 15.5 (6.8), 15, 12-22 | 0.87 |
| MFIS cognitive | 12.7 (9.3), 10, 4-24 | 13.7 (7.6), 12, 8-18 | 0.72 |
| MFIS psychosocial | 2.5 (2.3), 2, 0-5 | 2.1 (1.4), 2, 1-3 | 0.46 |
| MFIS total | 30.3 (20.2), 24, 13-49 | 31.3 (13.1), 31, 23-41 | 0.86 |
| Quality of life^‡^ | | | |
| EQ-5D index score | 0.61 (0.35), 0.73, 0.53-0.80 | 0.62 (0.35), 0.64, 0.50-0.80 | 0.97 |
| EQ-5D VAS | 71.6 (17.2), 75, 60-85 | 67.1 (18.0), 70, 50-80 | 0.44 |

**Table s1 legend.** EQ-5D, European quality of life-5 dimensions questionnaire; HADS, hospital anxiety and depression scale; MFIS, modified fatigue impact scale; MS, multiple sclerosis, MOGAD, myelin oligodendrocyte glycoprotein antibody-associated disease; SDMT, symbol digit modalities test; SPART, 10/36 spatial recall test; SPART-D, 10/36 spatial recall test delayed recall; SRT-D, selective reminding test delayed recall; SRT-CLTR, selective reminding test consistent long term retrieval; SRT-LTS, selective reminding test long term storage; VAS, visual analogue scale; WLG, word list generation.

^§^Data are presented as mean (standard deviation) of the Z-scores, number of patients with abnormal Z-score (i.e. < -1.5)/ number of patients with normal Z-score.

^†^Data are presented as mean (standard deviation), median, interquartile range, number of patients with abnormal corrected scores/number of patients with normal corrected scores.

^‡^Data are presented as mean (standard deviation), median, interquartile range.

* marks *p* < 0.05 for MS vs. MOGAD comparison.

Supplementary references

s16. Fisk JD, Ritvo PG, Ross L, Haase DA, Marrie TJ, Schlech WF. Measuring the Functional Impact of Fatigue: Initial Validation of the Fatigue Impact Scale. *Clinical Infectious Diseases*. 1994;18(Supplement_1):S79-S83. doi:10.1093/clinids/18.Supplement_1.S79

s17. Balestroni G, Bertolotti G. EuroQol-5D (EQ-5D): an instrument for measuring quality of life. *Monaldi Arch Chest Dis*. 2015;78(3). doi:10.4081/monaldi.2012.121

s18. Li X, Basso M, Chen J, Tillema JM, Pittock S, Flanagan E. Cognitive sequelae in MOG antibody-associated disease (P13-5.025). *Neurology*. 2023;100(17_supplement_2):3117. doi:10.1212/WNL.0000000000203034

s19. Kazzi C, Alpitsis R, O’Brien TJ, Malpas CB, Monif M. Cognitive and psychopathological features of neuromyelitis optica spectrum disorder and myelin oligodendrocyte glycoprotein antibody-associated disease: A narrative review. *Multiple Sclerosis and Related Disorders*. 2024;85:105596. doi:10.1016/j.msard.2024.105596

s20. Santoro JD, Gould J, Panahloo Z, Thompson E, Lefelar J, Palace J. Patient Pathway to Diagnosis of Myelin Oligodendrocyte Glycoprotein Antibody-Associated Disease (MOGAD): Findings from a Multinational Survey of 204 Patients. *Neurol Ther*. 2023;12(4):1081-1101. doi:10.1007/s40120-023-00474-9

s21. Petracca M, Pontillo G, Moccia M, et al. Neuroimaging Correlates of Cognitive Dysfunction in Adults with Multiple Sclerosis. *Brain Sciences*. 2021;11(3):346. doi:10.3390/brainsci11030346

s22. Jellinger KA. Cognitive impairment in multiple sclerosis: from phenomenology to neurobiological mechanisms. *J Neural Transm*. 2024;131(8):871-899. doi:10.1007/s00702-024-02786-y

s23. Tedone N, Preziosa P, Meani A, et al. Regional white matter and gray matter damage and cognitive performances in multiple sclerosis according to sex. *Mol Psychiatry*. 2023;28(4):1783-1792. doi:10.1038/s41380-023-01996-2

s24. Zhuo Z, Duan Y, Tian D, et al. Brain structural and functional alterations in MOG antibody disease. *Mult Scler*. 2021;27(9):1350-1363. doi:10.1177/1352458520964415

s25. Schneider R, Kogel A, Ladopoulos T, et al. Cortical atrophy patterns in myelin oligodendrocyte glycoprotein antibody‐associated disease. *Ann Clin Transl Neurol*. 2024;11(8):2166-2175. doi:10.1002/acn3.52137

s26. Chia NH, Redenbaugh V, Chen JJ, Pittock SJ, Flanagan EP. Corpus callosum involvement in MOG antibody-associated disease in comparison to AQP4-IgG-seropositive neuromyelitis optica spectrum disorder and multiple sclerosis. *Mult Scler*. 2023;29(6):748-752. doi:10.1177/13524585221150743

s27. Cacciaguerra L, Redenbaugh V, Chen JJ, et al. Timing and Predictors of T2-Lesion Resolution in Patients With Myelin Oligodendrocyte Glycoprotein Antibody–Associated Disease. *Neurology*. 2023;101(13). doi:10.1212/WNL.0000000000207478

s28. Marrie RA, Patel R, Bernstein CN, et al. Anxiety and depression affect performance on the symbol digit modalities test over time in MS and other immune disorders. *Mult Scler*. 2021;27(8):1284-1292. doi:10.1177/1352458520961534

s29. Nabizadeh F, Balabandian M, Rostami MR, et al. Association of cognitive impairment and quality of life in patients with multiple sclerosis: A cross-sectional study. *CJN*. Published online November 13, 2022. doi:10.18502/cjn.v21i3.11106
